# Genetic influences on plasma CFH and CFHR1 concentrations and their role in susceptibility to age-related macular degeneration

**DOI:** 10.1093/hmg/ddt336

**Published:** 2013-07-19

**Authors:** Morad Ansari, Paul M. Mckeigue, Christine Skerka, Caroline Hayward, Igor Rudan, Veronique Vitart, Ozren Polasek, Ana-Maria Armbrecht, John R.W. Yates, Zoran Vatavuk, Goran Bencic, Ivana Kolcic, Ben A. Oostra, Cornelia M. Van Duijn, Susan Campbell, Chloe M. Stanton, Jennifer Huffman, Xinhua Shu, Jane C. Khan, Humma Shahid, Simon P. Harding, Paul N. Bishop, Ian J. Deary, Anthony T. Moore, Baljean Dhillon, Pavao Rudan, Peter F. Zipfel, Robert B. Sim, Nicholas D. Hastie, Harry Campbell, Alan F. Wright

**Affiliations:** 1 MRC Human Genetics Unit, Institute of Genetics and Molecular Medicine, University of Edinburgh, Edinburgh EH4 2XU, UK,; 2 Centre for Population Health Sciences, University of Edinburgh, Edinburgh EH8 9AG, UK,; 3 Department of Infection Biology, Leibniz-Institute for Natural Product Research and Infection Biology, 07745 Jena, Germany,; 4 Croatian Centre for Global Health, University of Split, 21000 Split, Croatia,; 5 University of Split School of Medicine, 21000 Split, Croatia,; 6 Department of Ophthalmology, Princess Alexandra Eye Pavilion, University of Edinburgh, Edinburgh EH3 9HA, UK,; 7 Department of Medical Genetics, Cambridge Institute for Medical Research, University of Cambridge, CambridgeCB2 0XY, UK,; 8 Clinical Hospital ‘Sestre Milosrdnice’, Zagreb, Croatia,; 9 Department of Clinical Genetics,; 10 Department of Epidemiology and; 11 Department of Biostatistics, Erasmus University, 3000 CA Rotterdam, The Netherlands,; 12 Department of Ophthalmology, University of Liverpool, Liverpool L69 3BX, UK,; 13 School of Biomedicine, CMFT, Manchester Academic Health Sciences Centre, University of Manchester and Manchester Royal Eye Hospital, Manchester M13 9WL, UK,; 14 MRC Centre for Cognitive Ageing and Cognitive Epidemiology, Department of Psychology, University of Edinburgh, Edinburgh EH8 9JZ, UK,; 15 Institute for Anthropological Research, 10000 Zagreb, Croatia,; 16 Department of Infection, Immunity and Inflammation, University of Leicester, Leicester LE1 9HN, UK and; 17 Institute of Ophthalmology, University College London, London EC1V 9EL, UK

## Abstract

It is a longstanding puzzle why non-coding variants in the complement factor H (*CFH*) gene are more strongly associated with age-related macular degeneration (AMD) than functional coding variants that directly influence the alternative complement pathway. The situation is complicated by tight genetic associations across the region, including the adjacent *CFH*-related genes *CFHR3* and *CFHR1*, which may themselves influence the alternative complement pathway and are contained within a common deletion (CNP147) which is associated with protection against AMD. It is unclear whether this association is mediated through a protective effect of low plasma CFHR1 concentrations, high plasma CFH or both. We examined the triangular relationships of *CFH/CFHR3/CFHR1* genotype, plasma CFH or CFHR1 concentrations and AMD susceptibility in combined case–control (1256 cases, 1020 controls) and cross-sectional population (*n* = 1004) studies and carried out genome-wide association studies of plasma CFH and CFHR1 concentrations. A non-coding *CFH* SNP (rs6677604) and the CNP147 deletion were strongly correlated both with each other and with plasma CFH and CFHR1 concentrations. The plasma CFH-raising rs6677604 allele and raised plasma CFH concentration were each associated with AMD protection. In contrast, the protective association of the CNP147 deletion with AMD was not mediated by low plasma CFHR1, since AMD-free controls showed increased plasma CFHR1 compared with cases, but it may be mediated by the association of CNP147 with raised plasma CFH concentration. The results are most consistent with a regulatory locus within a 32 kb region of the *CFH* gene, with a major effect on plasma CFH concentration and AMD susceptibility.

## INTRODUCTION

Age-related macular degeneration (AMD) is the major cause of registered blindness in westernized countries, with 15–30 million people affected globally (1,2). There is currently no established means of preventing disease progression, although ocular injection of angiogenesis inhibitors in late neovascular AMD can halt or even reverse visual loss (3). Prospective studies show that progression to advanced stages of AMD is strongly predicted by risk genotypes in two genomic regions: complement factor H (*CFH*, OMIM #134370) and *ARMS2*/*HTRA1* (OMIM #611313) (4,5). The function of the *ARMS2/HTRA1* locus is unclear (6), but CFH is a strong suppressor of complement activation, acting at the level of C3b (7,8). CFH inhibits alternative pathway activation both in plasma and on cell surfaces by promoting C3b proteolysis and either preventing the formation or enhancing the dissociation of C3 convertases. This pathway is strongly implicated in AMD risk (9), since several components are either present in diseased tissues, such as the characteristic extracellular drusen deposits (CFH, C3b/iC3b, BF, C5b-9), or they are genetically associated with disease risk (*C3*, *C2*/*BF*, *CFH*, *CFHR1*, *CFHR3*, *CFI*) (10,11). Mutations in *CFH* are also implicated in the renal disorders type II membranoproliferative glomerulonephritis and atypical haemolytic–uraemic syndrome (aHUS) (8), and one rare variant is implicated in causing both AMD and aHUS (12).

The *CFH* gene encodes two protein products, CFH and factor H-like 1 (FHL-1) by alternative splicing. The 7 short consensus repeats (SCRs) of FHL-1 are identical to the N-terminal part of CFH, which has 20 SCRs. FHL-1 also differs from CFH because of an additional four amino acids at its C-terminus (SCR7) and the availability of an RGD motif in SCR4 associated with adhesive activity (13). The molar concentration of FHL-1 in plasma is only 10–30% of plasma CFH levels (14).

The structurally similar CFH-related proteins CFHR1 and CFHR3 are also less abundant than CFH in plasma and are encoded by genes immediately adjacent to *CFH* within the Regulator of Complement Activation (RCA) gene cluster in chromosomal region 1q31.3. CFHR1 and CFHR3 have also been proposed to influence both aHUS and AMD susceptibility via effects on complement activity (15,16). CFHR1 inhibits C5 convertase activity and terminal membrane attack complex (MAC) formation, whereas CFHR3 inhibits C3 convertase activity *in vitro* (16,17). CFHR1, CFHR3 and CFH show considerable sequence homology in their C-terminal SCR modules, which have been proposed to compete for binding to C3b and heparin, consistent with a regulatory role (16,17). Homozygosity for a common 86 kb *CFHR3*/*CFHR1* (CNP147) deletion, which has an allele frequency of 0.2–0.3 in Western populations, is associated with increased risk of aHUS, suggesting that one or both proteins act together to suppress MAC formation and inflammation (16). But, in AMD, the situation is reversed, since CNP147 homozygosity is associated with protection against macular degeneration (18), which has been explained by the beneficial effects of enhanced opsonin (iC3b, C5a) formation, promoting phagocytosis of opsonized particles in AMD due to the reduced inhibition of the C5 convertase (15,17,19). However, interpreting these genetic associations is complicated by the presence of strong linkage disequilibrium (LD) across the region. Hughes *et al*. (18) initially proposed that the CNP147 deletion acts independently of non-synonymous coding variants in *CFH*, such as Y402H, which affect CFH function (20–22). Subsequent analyses, however, showed that the strongest associations with AMD across the *CFH*-*CFHR3*-*CFHR1* region were independent of the CNP147 deletion (23,24) and resulted from non-coding or synonymous variants lying within a small 32 kb region in the *CFH* gene between rs203687 in intron 9 and rs1329427 in intron 15 (25).

In order to try and resolve these issues, we measured the triangular relationships between *CFH-CFHR3-CFHR1* genotypes, plasma concentrations of CFHR1 and CFH plus FHL-1 (hereafter referred to as ‘CFH’) and AMD susceptibility, using both cross-sectional population cohort (*n* = 1004) and AMD case–control series (1256 cases, 1020 controls) (see Supplementary Material, Table S1 and Fig. S1). We identified a locus within the *CFH* gene with a substantial effect on both plasma CFH and CFHR1 concentrations, consistent with the stronger association of AMD with non-coding rather than non-synonymous coding *CFH* SNPs. We also found that raised plasma CFH concentration and the presence of a plasma CFH-raising allele at *CFH* SNP rs6677604 were each associated with AMD protection. On the other hand, whereas the CNP147 deletion was associated with AMD protection, measured plasma CFHR1 was unexpectedly lower in AMD cases than controls, excluding the possibility that disease protection by CNP147 is mediated by reduced plasma CFHR1. Instead, the association of CNP147 with raised plasma CFH may be the major protective influence, whereas the low CFHR1 concentration in AMD cases appears to be due to confounding influences, such as the effect of disease *per se*.

## RESULTS

### Genotypic effects on plasma CFH and CFHR1 concentrations

Plasma CFH was normally distributed with a 3-fold range (218–654 μg/ml) and a mean of 400 ± 62 (SD) μg/ml in a Croatian (Vis) cross-sectional population sample (*n* = 1004) (Supplementary Material, Fig. S2 and Table S2). Plasma CFHR1 concentrations, expressed as a fraction of the reference samples used in the enzyme-linked immunosorbent assay (ELISA), were measured in the same samples and were also normally distributed, with a mean of 0.93 ± 0.20 (SD) standardized units and a range of 0.19–1.55 (Supplementary Material, Fig. S2 and Table S2). To exclude possible cross-reactivity between the two assays, we examined the correlation between plasma CFH and CFHR1 concentrations, using the residuals from linear regressions of each analyte on age, sex and genotype. Adjustment for age, sex and genotype is appropriate because to detect cross-reactivity we want to eliminate associations between CFH and CFHR1 concentrations that are explained by covariates. We evaluated the posterior distribution of the residual correlation in the Bayesian linear regression model used for the instrumental variable analysis (see below): this yields point and interval estimates (posterior mean and 95% credible interval) that allow for missing genotypes and phase uncertainty. The correlations between residuals of plasma CFH and CFHR1 concentrations were 0.01 (95% CI −0.02 to 0.04) in the Scottish AMD (SAMD) sample and −0.05 (95% CI −0.11 to 0.00) in the Croatian samples, clearly showing that any cross-reactivity between the two assays is minimal.

We took advantage of the genetic relationships within the Croatian isolate population to estimate the heritability of plasma CFH and CFHR1 concentrations. Narrow-sense heritability, which is the proportion of the total phenotypic variance due to additive genetic variance, was estimated to be 0.46 ± 0.13 (SEM) for plasma CFH and 0.69 ± 0.12 (SEM) for plasma CFHR1 concentration in the Croatian population, consistent with a substantial heritable component for both biomarkers.

**Figure 1.**
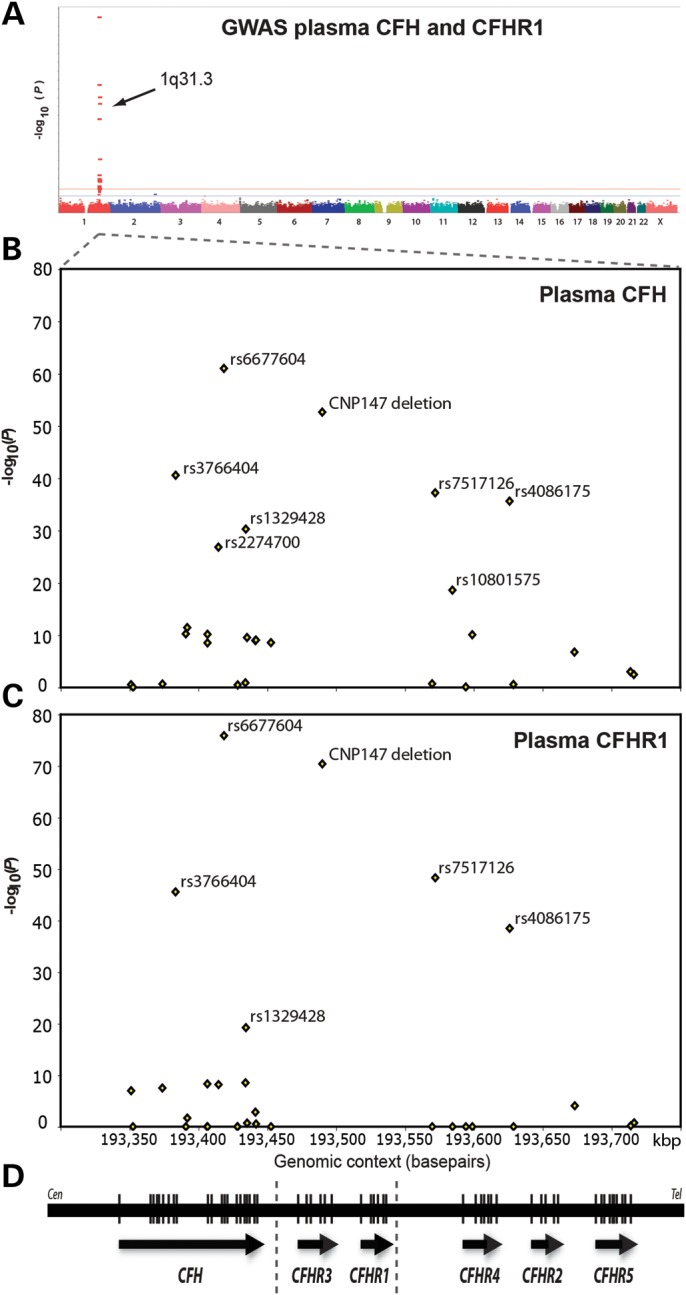
Results of GWAS of plasma CFH and CFHR1 in a normal population cohort. Variants in the *CFH* gene and the CNP147 deletion show the strongest genetic associations with both plasma CFH and plasma CFHR1 concentrations. (**A**) Manhattan plot summarizing GWAS results of plasma CFH in a Croatian cross-sectional population cohort (*n* = 1004) showing transformed (−log_10_) *P*-values for all SNPs used in the study. A similar plot for plasma CFHR1 is shown in Supplementary Material, Figure S3. (**B**) GWAS results using a full mixed model showing chromosomal region 1q32 in more detail: the locations and genetic association *P*-values for 27 SNPs in the *CFH* to *CFHR5* genomic region are shown. (**C**) The corresponding results for the plasma CFHR1 GWAS (*n* = 1004). (**D**) Genomic annotations showing the boundaries of the *CFHR3*/*CFHR1* (CNP147) deletion (dotted vertical lines), direction of transcription (arrows) and genomic location of exons (to scale, vertical lines within the genes).

**Figure 2.**
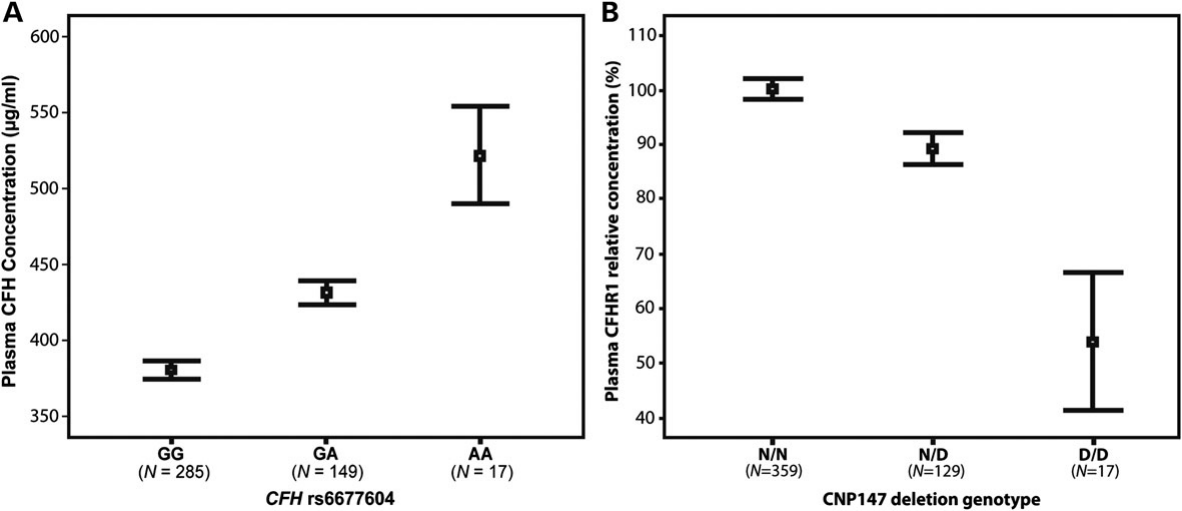
Effects of *CFH* SNP rs6677604 and the CNP147 deletion on plasma CFH and CFHR1 concentrations, respectively. (**A**) Means and 95% confidence intervals of plasma CFH in the Croatian population cohort showing the association with rs6677604 SNP genotypes (GG, GA, AA). The minor allele (A) is associated with an increase in plasma CFH under an additive model [*r* = 0.52, *P* = 2.9 × 10^−33^ (two-tailed, *n* = 451)]. Error bars are 95% confidence intervals of the mean (black squares). *n* is the number of unrelated individuals in each group. (**B**) The CNP147 (*CFHR3*/*CFHR1*) deletion (D) genotype and plasma CFHR1 concentrations are correlated in the Croatian cross-sectional population cohort. As the CNP147 copy number changes from 0 to 2, plasma CFHR1 concentrations are reduced accordingly [*r* = −0.62, *P* = 4.3 × 10^−42^ (two-tailed, *n* = 505)]. Error bars are 95% confidence intervals of the mean (black squares). A similar result was obtained in the SAMD series (see text).

In order to identify genetic variants influencing plasma CFH concentration, a genome-wide association study (GWAS) was carried out in the Croatian population cohort (*n* = 1004), as described previously (26) using 317 503 SNPs (Supplementary Material, Fig. S3). The SNP most strongly associated with plasma CFH concentration was rs6677604 in *CFH* intron 11 (Fig. 1), with each minor (A) allele increasing CFH concentration by 25.6 µg/ml (SEM 1.6) (*P* = 1.1 × 10^−61^) (Fig. 2A and Supplementary Material, Table S3). This SNP explained 26% of the measured variance in plasma CFH concentration (57% of additive genetic variance), adjusted for covariates age, sex and BMI. The result was replicated in 500 unrelated individuals from a Dutch (Rucphen) cross-sectional population cohort (Supplementary Material, Tables S1 and S2) (27), in which rs6677604 was again the most strongly associated SNP [*β* = 41.5 µg/ml (SEM 3.9), *P* = 6 × 10^−24^], using the same covariates (Supplementary Material, Table S4).

A GWAS was also carried out to identify genetic variants influencing plasma CFHR1 concentration in the same Croatian population cohort. The strongest genotypic association of plasma CFHR1 was again found for *CFH* SNP rs6677604, at which the minor (A) allele decreased plasma CFHR1 concentration [*β* = −0.23 ± 0.01 (SEM), *P* = 1.31 × 10^−76^], and with the CNP147 deletion, which was associated with reduced plasma CFHR1 [*β* = −0.22 ± 0.01 (SEM), *P* = 3.98 × 10^−71^] (Fig. 1, Supplementary Material, Fig. S3 and Table S3). The rs6677604 minor (A) allele is highly correlated with the CNP147 deletion (*r*^2^ = 0.84) (Supplementary Material, Table S5) and this locus explained 40% of the measured variance in plasma CFHR1 concentration (58% of additive genetic variance), adjusted for age, sex and BMI in the regression model.

The plasma concentration of CFHR1 was inversely correlated with the CNP147 deletion genotype [*r* = −0.62, *P* = 4.3 × 10^−42^ (Croatians) and *r* = −0.43, *P* = 1.2 × 10^−23^ (SAMD)] (Fig. 2B). Analysis of LD in the unrelated individuals from the Croatian data set showed that the minor allele (A) of *CFH* SNP rs6677604 was only weakly correlated with the minor alleles of two non-synonymous SNPs that have previously been associated with AMD risk, in exon 2 [rs800292 (I62V), T-allele, *r*^2^ = 0.04] (28) and exon 9 [rs1061170 (Y402H) C-allele, *r*^2^ = 0.16] (20–22).

**Figure 3.**
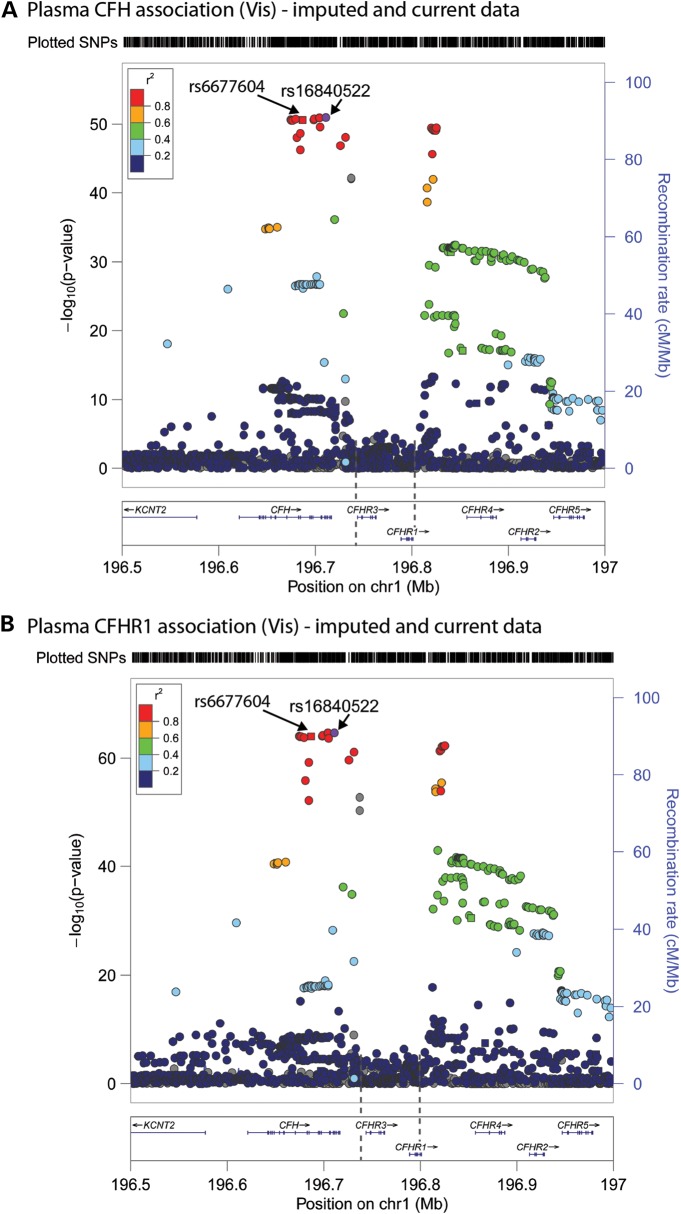
GWAS of plasma CFH and CFHR1 concentrations in the Croatian cohort (*n* = 1004), including both genotyped and imputed SNPs. Manhattan plots showing GWAS results for plasma CFH (**A**) and CFHR1 (**B**) using 1000 Genomes Project imputed data, in which the circles show the statistical strength of the association (−log_10_ *P*-value), coloured according to the pair-wise correlations (*r*^2^) between surrounding markers and the most strongly associated variant (imputed SNP rs16840522). Variants that were poorly imputed (imputation Rsq ≤ 0.4) were filtered out and the results are shown for a 500 kb window encompassing the *CFH* and *CFHR1-5* genes. The right-hand *y*-axis and purple line show recombination rates across the region, whereas the *x*-axis shows the position on chromosome 1 and genomic annotations showing the boundaries of the *CFHR3*/*CFHR1* (CNP147) deletion (dotted vertical lines), direction of transcription (arrows) and genomic location of exons (to scale, vertical lines within the genes).

In order to examine the effects of additional variants in this region, we repeated the GWAS for both plasma CFH and CFHR1 on 1000 Genomes Project imputed data (http://www.1000genomes.org/ last accessed July 2013) after adjusting for age, sex, age-by-sex and body mass index and using a score test for association (mmscore) in related individuals, as implemented in the GenABEL suite (29,30). Variants which were poorly imputed (Rsq ≤ 0.4) were filtered out. The analysis focused on a 500 kb window encompassing the *CFH*- and *CFH*-related genes (Fig. 3). Furthermore, we assessed the LD between the imputed top SNP (rs16840522) and our top SNP (rs6677604), using SNAP (http://www.broadinstitute.org/mpg/snap last accessed July 2013). These two SNPs are 24 kb apart, with an *r*^2^ = 0.98 and *D′* = 1.00, showing that they are in very high LD. Both SNPs lie within the 32 kb region of *CFH* referred to above. We repeated the plasma CFH and CFHR1 association analysis adjusting for our original top SNP (rs6677604). The peaks for all the SNPs in LD with rs16840522 disappeared, suggesting that the same signal had been picked up in our original GWAS as with the imputed GWAS (Supplementary Material, Fig. S4).

### Association between plasma CFH and CFHR1 concentrations and AMD

**Figure 4.**
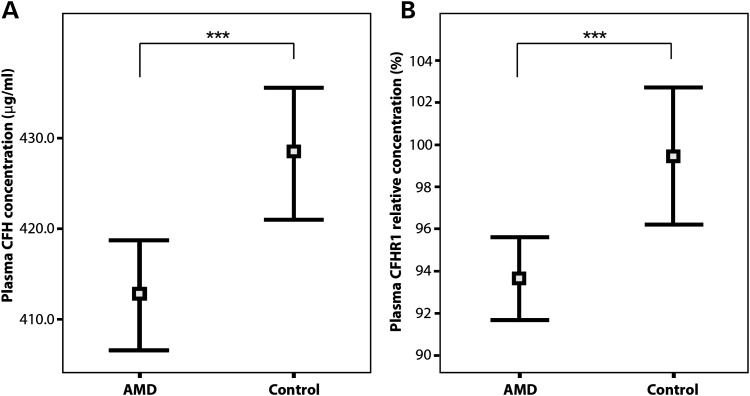
Comparison of plasma CFH and CFHR1 concentrations in the SAMD series. Unadjusted plasma CFH (**A**) and CFHR1 (**B**) concentrations in 382 AMD cases and 201 disease-free controls. The mean and 95% confidence intervals are shown. The differences in mean value were statistically significant for plasma CFH and CFHR1 after adjusting for age, sex and smoking status. ***P < 0.001, using a *t*-test (two-tailed).

We next examined the relationships between plasma CFH or CFHR1 and disease. The odds ratios for the effect of plasma CFH and CFHR1 concentrations on AMD risk were computed by logistic regression using data from a previously described SAMD case–control series (31) including 382 cases and 201 controls (Supplementary Material, Table S1 and Fig. S1). The mean unadjusted plasma CFH concentration was 15.7 μg/ml (0.27 standard deviations) lower in cases [412.6 ± 2.98 (SEM) μg/ml] than controls [428.3 ± 3.66 (SEM) μg/ml] (*P* = 0.001) (Fig. 4, Supplementary Material, Table S2). This difference remained significant after adjusting for age and sex (*P* = 0.002). The odds ratio for a plasma CFH increase of 1 standard deviation on AMD risk was estimated to be 0.52 (95% CI 0.37–0.70).

The mean unadjusted plasma CFHR1 concentration was also lower (by 0.28 standard deviations) in SAMD cases [0.94 ± 0.009 (SEM) standardized units] compared with controls [0.99 ± 0.016 (SEM) standardized units] (*P* = 0.001). The difference remained significant after adjusting for age and sex (*P* = 0.002) (Fig. 4, Supplementary Material, Table S2). This result was surprising since previous genotyping studies (18) suggested a protective effect of the CNP147 deletion on AMD risk, and the deletion allele was associated with decreased plasma CFHR1 concentration (Fig. 2B). AMD cases were, therefore, expected to show higher plasma CFHR1 than controls. Instead, increased plasma CFHR1 was protective for AMD, after adjusting for sex, age and plasma CFH concentration [odds ratio for the effect of a 1 standard deviation increase in plasma CFHR1 on AMD risk was 0.72 (95% CI 0.59–0.87; *P* = 0.001)]. The protective association between the CNP147 deletion and AMD risk is, therefore, not mediated by its effect on lowering plasma CFHR1 concentration.

In order to exclude the possibility that the plasma CFH or CFHR1 differences between cases and controls are due to the age-dependence of plasma CFH (and potentially CFHR1) concentrations or to smoking status (32), we repeated the analyses after additionally adjusting for age, sex and smoking status, but did not find substantial differences.

The OX23 monoclonal antibody (33) used in the CFH assay does not cross-react with CFH-related proteins (CFHR1-5) but does detect the CFH isoform, FHL-1. In order to investigate whether plasma FHL-1 also differs between AMD cases and controls, we carried out western blot analysis on plasma from five individuals with high plasma CFH concentrations and five individuals with low plasma CFH concentrations as previously determined by ELISA. In western blots, the OX23 antibody did not perform well, so the monoclonal murine anti-human CFH antibody (Quidel, clone 90X) was used, which binds specifically to SCR-1 on both CFH and factor H-like proteins (34) (Supplementary Material, Fig. S5). The blot images were analysed by densitometry, which showed that, even in such a small sample, differences could be seen in plasma CFH, but FHL-1 concentrations were comparatively small (molar concentration 1.8–8.1% of the CFH peak) and did not differ significantly between high and low CFH samples. The major association signal appeared, therefore, to be due to CFH rather than FHL-1.

### Genetic effects at the *CFH-CFHR1-CFHR3* loci on AMD susceptibility

**Figure 5.**
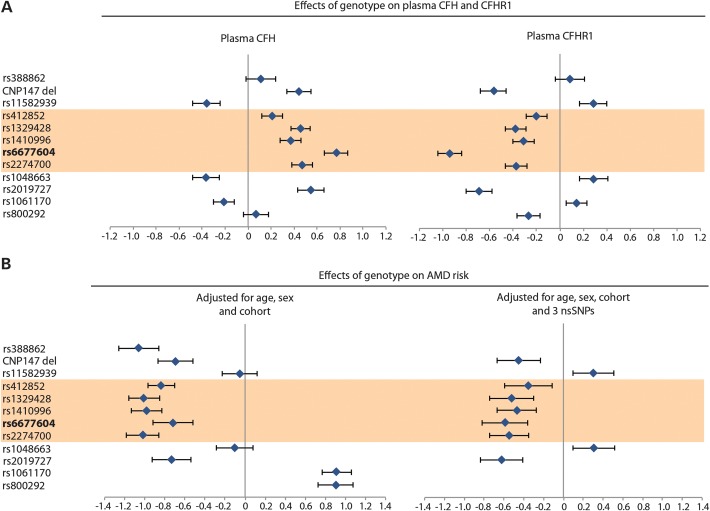
Univariate analysis predicts that SNPs located within a 32 kb region of the *CFH* gene have strong (but opposite) effects on plasma CFH and CFHR1 concentration. (**A**) Results of univariate logistic regression analysis showing the direction and magnitude of minor allele effect sizes of 12 *CFH*/*CFHR1* genetic variants influencing plasma CFH, plasma CFHR1 and AMD risk. Univariate linear regression coefficients are shown for the effects of minor allele genotype on plasma CFH and CFHR1, adjusted for age and sex, based on a combined analysis of SAMD control and Croatian samples. SNPs lying within a 32 kb region of *CFH* proposed by Sivakumaran *et al*. (25) to influence AMD risk are highlighted in orange. (**B**) Univariate logistic regression coefficients for the effects of genotype on AMD risk, adjusted for age, sex and cohort (left panel) or age, sex, cohort and three non-synonymous SNPs in CFH and CFHR1 (right panel), based on the combined analysis of SAMD and EAMD case–control series.

For the analysis of genetic effects on AMD risk, two case–control series were combined: the SAMD case–control series (31), which included a subset of 242 advanced AMD cases (atrophic or neovascular type) and 201 controls in whom both plasma CFH and CFHR1 had been measured, and an English AMD (EAMD) case–control series with 874 advanced AMD cases and 418 controls (31) (Supplementary Material, Table S1 and Fig. S1). The analysis focused on 12 genetic variants—11 SNPs and the CNP147 deletion (Fig. 5). These SNPs were chosen because they fulfilled one or more of the following criteria: (i) they encoded non-synonymous and potentially functional coding changes in *CFH* (rs1061170, rs800292) or *CFHR1* (rs388862); (ii) they were strongly associated with plasma CFH or CFHR1 concentration in the GWAS, including non-coding SNPs rs2019727, rs1048663, rs6677604, rs1329428, rs412852, rs11582939 and the CNP147 deletion (Fig. 1); (iii) they were the SNPs most strongly associated with AMD in other studies (rs2274700, rs1410996) (23,24).

The association of *CFH/CFHR3/CFHR1* SNPs with AMD was first examined by univariate logistic regression analysis, adjusting for age and sex. The magnitude and the direction of the effect of the 12 variants on AMD risk are shown in Figure 5,where they are also compared with their effects on plasma CFH or CFHR1, as determined in the SAMD case–control series and the Croatian population cohort. BMI was not measured in the SAMD or EAMD studies and so was not used as a covariate in this analysis. The results showed, first, that for many but not all SNPs, the genotypic effects on AMD risk were consistent with their effects on plasma CFH, in which increased plasma CFH was protective against AMD (Supplementary Material, Fig. S6).

Two additional observations were made. First, the CNP147 deletion was associated both with reduced plasma CFHR1 (*P* = 1.2 × 10^−23^) and with increased plasma CFH concentrations (*P* = 1.3 × 10^−6^) after adjusting for sex and age. This suggests an alternative mechanism whereby the CNP147 deletion could be associated with protection from AMD—through the association of CNP147 with raised plasma CFH, which was consistently protective against AMD, based both on genotype predictions and direct measurements (Fig. 4). Second, for those five *CFH* SNPs located within the 32 kb region reported to show the strongest association with AMD (25) (highlighted in orange in Fig. 5), the minor alleles were each associated with increased plasma CFH and lower AMD risk. The direction of SNP effects on plasma CFH is shown for their minor alleles, and the same alleles are consistently associated with opposite effects on plasma CFHR1 concentration (Fig. 5A). In contrast, *CFH* SNPs that alter the sequence and potentially the function of the protein [rs1061170 encoding Y402H (20–22,35,36) and rs800292 encoding I62V (28,35,37)] have effects on plasma CFH that appear uncorrelated with their effects on AMD risk (Fig. 5). For example, the above two non-synonymous *CFH* SNPs are associated with either no effect or a marginally lower plasma CFH, whereas both show a substantially increased AMD risk. Similarly, these SNP alleles are associated with either raised or lowered plasma CFHR1 and yet both are associated with a substantially increased AMD risk. This applies also to the minor allotype of complex SNP rs388862 (see Materials and Methods), which alters the protein sequence of CFHR1 (38) but has no significant effect on either plasma CFH or CFHR1 and yet is associated with a substantially lower AMD risk [Fig. 5 and (39)]. We, therefore, adjusted for these three non-synonymous SNPs as additional covariates in the regression model and re-examined the univariate effects of the remaining nine loci on AMD risk (Fig. 5B, right-hand caption). The resulting genotype-disease associations remained consistent with the measured plasma CFH concentrations, suggesting that, after accounting for functional SNP effects, high plasma CFH had a protective effect on AMD susceptibility, although an effect of low plasma CFHR1 could not be excluded. A multivariate analysis was, therefore, carried out to examine their relative contributions in more detail.

### Inference of causal effects due to plasma CFH and CFHR1 on AMD susceptibility

The results discussed above all involved combining different study data sets, all of which (SAMD, EAMD, Croatian) were genotyped across the RCA locus but only some of which had measurements of plasma CFH or CFHR1 (SAMD, Croatian). The resulting triangular associations (Supplementary Material, Fig. S6) were examined in a multivariate analysis in order to indicate causal effects or otherwise of plasma CFH and CFHR1 concentration on AMD susceptibility. This was done by means of an instrumental variable (‘Mendelian randomization’) analysis (see Materials and Methods and Supplementary Material, for further description). We used a Bayesian model since this can readily deal with missing data and multiple data sets. The analysis combined genotypic, biomarker (plasma CFH/CFHR1) and AMD data from the SAMD, EAMD and Croatian populations in a single model, bringing together the relationships between each point in the triangle, as discussed above.

The results of the analysis indicated a causal protective effect of plasma CFH on AMD risk, with an odds ratio for an increase of 1 standard deviation in plasma CFH concentration of 0.52 (95% CI 0.37–0.70), based on the posterior distribution of the log odds ratio. The corresponding causal odds ratio for plasma CFHR1 concentration on AMD risk was not significantly different from the null value of 1, with an odds ratio of 1.32 and 95% CI value of 0.88–2.08. Functional SNPs in *CFH* or *CFHR1* are likely to have effects on AMD that are not mediated by plasma CFH or CFHR1 (pleiotropic effects) and so were included as covariates in the regression model. The direct effects of the two non-synonymous *CFH* SNPs on AMD risk were either protective (rs800292 minor allele) [(odds ratio for one extra copy of the minor allele was 0.47 (95% CI 0.36–0.62)] or increased risk [odds ratio for one extra copy of the minor allele was 1.69 (95% CI 1.33–2.13)]. There was no evidence of a direct effect of the *CFHR1* nsSNP rs388862 on AMD risk (odds ratio 0.96, 95% CI 0.69–1.34). These results, first, emphasize the importance of accounting for the pleiotropic effects of the non-synonymous *CFH* SNPs on AMD risk in the model. Second, the results were consistent with a causal effect of lower plasma CFH (and only a possible effect of raised plasma CFHR1) on AMD risk.

## DISCUSSION

We show that variation in the *CFH* gene, at sites other than the two potentially functional nsSNPs, I62V and Y402H, is associated with large effects on plasma CFH concentration. The most strongly associated SNP is rs6677604 in *CFH* intron 11, which accounts for about half of the genetic variance in plasma CFH in a univariate analysis. Our results are consistent with a causal regulatory locus within a 32 kb region of the *CFH* gene, which includes rs6677604 (25). The nature of this locus remains unknown, since no candidate eQTL maps to the region of rs6677604 and we were unable to identify enhancer activity using luciferase reporter assays, although the region remains to be comprehensively screened. No locus outside the RCA gene cluster in chromosomal region 1q32 was evident in the GWAS of plasma CFH or of CFHR1 (Supplementary Material, Fig. S3).

Plasma CFH concentration varied by a factor of 3 in our population sample and showed high heritability, as reported in other populations (32). The plasma CFH concentration measured by our ELISA assay included both CFH and its alternatively spliced isoform FHL-1; so, we cannot discriminate between these isoforms, although we confirmed that CFH is expressed at over 10-fold higher molar concentration in plasma compared with FHL-1 [Supplementary Material, Fig. S5 and (40)]; so, FHL-1 is unlikely to influence the reported genetic associations substantially. Also, CFH and CFHR1 concentrations in the Croatian population cohort were found to be inversely correlated, with a Pearson correlation coefficient of −0.34 (95% CI −0.40 to −0.28) (*P*-value < 2.2 × 10^−16^) after adjusting for covariates, suggesting that although the ELISAs are measuring different proteins, loci influencing their expression levels are in some degree of LD, which spans most of the RCA region (see Supplementary Material, Table S5).

Could the proposed locus influencing plasma CFH explain the effect of the CNP147 deletion on AMD risk as a result of LD across the region? The CNP147 deletion was in LD with CFH-raising alleles at rs6677604 (*r*^2^ = 0.84) and rs2274700 (*r*^2^ = 0.33), a synonymous coding SNP (A473A) in exon 10 of *CFH* (24), both of which are located within a 32 kb region of the *CFH* gene that shows the strongest association with AMD (23–25). Fritsche *et al*. (16) found evidence for the conditional independence of the CNP147 deletion from *CFH* SNPs rs2274700 (Ala473Ala) and rs1061170 (Y402H) and their effects on AMD risk. In contrast, Raychaudhuri *et al*. (41) used a larger panel of *CFH* SNPs and found that the protective effect of CNP147 was not independent of *CFH* haplotypes. We concur with this result, since we found strong LD between rs6677604 and CNP147, suggesting that their effects are likely to be hard to separate genetically.

As the CFH-raising minor (A) allele at *CFH* SNP rs6677604 is strongly correlated with the CNP147 deletion, the association of this deletion with lower AMD risk is compatible with a protective effect of high plasma CFH concentration or of low plasma CFHR1 concentration (or both). It has been suggested that low CFHR1 concentration (predicted by genotype but not previously measured) protects against AMD by reduced competitive inhibition of CFH binding to tissue surfaces, leading to greater inhibition of the C3 convertase by CFH and consequently enhanced protection against inflammation (16). Alternatively, the genetically predicted reduction in plasma CFHR1 may increase C5 convertase and MAC formation, the former leading to enhanced opsonization of particles, which may be advantageous in AMD (but not aHUS). A further possibility is that the two proteins, CFH and CFHR1, act sequentially on the alternative pathway C3 and C5 convertases, consistent with the observed competition for C3b binding and their presence together in human macular and choriocapillary tissues. Although the plasma concentration of CFH is in substantial molar excess over CFHR1 (Supplementary Material, Fig. S7), it remains possible that they act jointly to suppress complement activation at different steps in the alternative pathway.

We have measured plasma CFHR1, for the first time, in AMD cases and controls, in addition to plasma CFH (Fig. 4). The CNP147 deletion genotype was associated with low plasma CFHR1, as expected (Fig. 2B), and was protective against AMD, as reported by others (Fig. 5). However, the plasma CFHR1 concentration was lower in AMD cases than controls (Fig. 4) despite the fact that the frequency of the deletion is lower and the expected level, given genotype, is higher in cases than in controls. This was initially surprising, although a low CFHR1 level might enhance inflammatory processes by reduced inhibition of successive steps in the alternative pathway. However, since we effectively excluded the possibility of a protective association between AMD and low plasma CFHR1 (Fig. 4), the most likely explanation is the strong association of the CNP147 deletion with raised plasma CFH concentration which provides protection against AMD.

In the renal disease aHUS, the presence of a homozygous CNP147 deletion is associated with increased disease risk, whereas in AMD it is associated with reduced risk. A possible explanation for this discrepancy is that, in AMD, this effect is due to LD between the CNP147 deletion and *CFH* variants, whereas the deletion (and low plasma CFHR1/CFHR3) may be directly causal in aHUS, in which the complement pathology differs in several respects from AMD (8).

The measurement of plasma CFH and CFHR1 concentrations in AMD cases was made after disease onset, so it is possible that their concentrations may have been influenced by the disease process. Mean plasma CFH concentrations were lower in the 382 SAMD cases than in 201 AMD-free controls, similar to the findings of Reynolds *et al*. (42), who studied 120 advanced AMD cases and 60 controls and found a small but statistically significant plasma CFH reduction in AMD cases (*P* = 0.009). Two other studies, by Hakobyan *et al*. (43) (53 cases/75 controls) and Scholl *et al*. (44) (112 cases/67 controls), did not find statistically significant associations. However, none of these studies measured both CFH and FHL1 in plasma and all of them were under-powered, with only 31–42% power to detect (*P* < 0.05) the observed case–control difference in plasma CFH of 0.27 standard deviations that we observed (for which our study had 84% power).

Non-synonymous *CFH* and *CFHR1* coding variants have functional effects on AMD risk that appear to act independently of plasma CFH or CFHR1concentrations. For example, reduced binding to polyanionic surfaces has been found with the 402H allele compared with 402Y (21,22). In the univariate analyses (Fig. 5), many non-coding *CFH* SNPs showed stronger associations with AMD risk than the three coding (non-synonymous) SNPs, as observed by others (23,24). The strongest univariate SNP associations with AMD risk are likely to be with those SNPs that are in LD with both the non-synonymous coding variants and the proposed locus regulating plasma CFH concentration (near rs6677604).

There are as yet no clinical data on the effect of raising plasma CFH concentrations therapeutically but we would hypothesize that raising plasma CFH concentration by 1 standard deviation will approximately halve the incidence of AMD, based on the results of the instrumental variable analysis, in which all the data were examined together. However, any substantial increase in plasma CFH would be hard to achieve in practice due to its high plasma concentration, even if its half-life in plasma could be extended. In individuals whose genotype encodes an apparently less functionally active form of CFH (e.g. 402H homozygotes), the effect of raising the concentration of the presumed more active form (402Y) may, however, be greater than in the general population. For therapeutic use of CFH to be feasible, it would also be necessary to develop screening tests either for the prediction of disease risk or for early diagnosis, and to develop a convenient means of delivering the protein. A prospective cohort study of genotype- and phenotype-based AMD risk scores in a population of European ancestry showed a 19-fold range of predictive risk with a discriminative ability [concordance (C) statistic = 0.78] similar to that of Framingham risk scores for coronary heart disease (5). Inclusion of non-genetic and genetic factors in a validation sample gave a similar C statistic of 0.809 for progression to advanced AMD at 10 years (45). Targeting of therapy to high genetic risk individuals may, therefore, be feasible. The major source of plasma CFH is the liver, but extra-hepatic tissues, including retinal pigment epithelium and glomerular mesangial cells, also contribute (7,8). Therapeutic use of CFH delivered by 2-weekly intravenous injection of fresh frozen plasma (half-life 6 days) (46) is not practicable on the necessary scale, but local delivery of an inducible recombinant CFH to the choroid or retinal pigment epithelium by a viral vector may be more achievable (47,48). The alternative of CFH gene delivery to the liver (49) to boost plasma concentrations raises significant risk–benefit issues but may become feasible in the future.

## MATERIALS AND METHODS

### Subject recruitment

Subject recruitment has been described for the Croatian (26), Dutch (Rucphen) (27), SAMD (31) and EAMD (31) samples. The sample sizes, AMD status and contributions of each series or cohort to the present study are summarized in Supplementary Material, Table S1.

### Plasma CFH assay

An ELISA was developed to measure plasma CFH. Blood samples were collected in EDTA-anticoagulated tubes. Ninety-six-well microtitre plates (Greiner Bio-One) were coated with 50 μl of 1.2 μg/ml sheep polyclonal anti-CFH capture antibody (Abcam), diluted in 0.1 m glycine, pH 9.5. After 2 h of incubation at room temperature, the plates were washed four times in 137 mm NaCl, 10 mm phosphate, 2.7 mm KCl, pH 7.4 (PBS) and 0.05% Tween. An amount of 100 μl of blocking solution [1% bovine serum albumin (BSA, 98% lyophilized powder) in dH_2_O] was added and further incubation carried out at room temperature, for at least 2 h. Doubling dilutions of the 1.5 mg/ml CFH standard were carried out from a starting concentration of 150 ng/ml, diluted in PBS containing 0.1% BSA and 0.05% Tween. The CFH standard was prepared according to the method of Sim *et al*. (50). Fifty microlitres of each sample, diluted 1 in 5000, plus standard and internal control samples were added to the plate wells, in triplicate. Samples were incubated in plates coated with the capture antibody for 2 h at room temperature. Fifty microlitres of the 85 ng/ml mouse monoclonal anti-CFH (MRCOX23) detection antibody (33) which specifically detects CFH and FHL-1 (not CFHR proteins) was added to each well, followed by a 3 h incubation at room temperature. After four washes, 50 μl of the 145 ng/ml horseradish peroxidase (HRP)-conjugated goat anti-mouse IgG antibody (Biosource UK) was added to each well and incubated for 30 min at room temperature. Plates were then washed four times and developed after addition of 100 μl of 3,3′,5,5′-tetramethylbenzidine chromogen (Biosource UK) and incubation for 1 h at room temperature. Enzymatic reactions were terminated using 100 μl of 0.5 m sulphuric acid and the absorbance read at 450 nm using the SkanIt plate reader (Thermo Electron). Internal controls were used to normalize inter-experimental variation.

### Plasma CFHR1 assay

Plasma CFHR1 concentration was determined by ELISA assay using mouse monoclonal antibody JHD10 (17) (diluted 1 in 1000), which was immobilized on a MaxiSorp microtitre plate, and non-specific binding sites were blocked with 2% BSA in PBS, 0.05% Tween. The plates were incubated with plasma (diluted 1 in 50, using 2% BSA in PBS, 0.05% Tween) for 1 h at room temperature. Plates were washed and bound protein was detected using a rabbit polyclonal anti-CFHR1 antibody (51) (diluted 1 in 4000 using 2% BSA in PBS, 0.05% Tween). Secondary detection was carried out using an HRP-conjugated mouse anti-rabbit IgG antibody (diluted 1 in 4000 as before). OPD (*o*-phenylenediamine dihydrochloride, Sigma, Germany) was added and absorbance measured at 490 nm after addition of stop solution. For each microtitre plate, CFHR1 concentrations were also determined in duplicate plasma controls, derived from one individual who is genetically identified as homozygous *CFHR3*/*CFHR1* sufficient (positive control) and one individual who is homozygous *CFHR3*/*CFHR1* deficient (negative control). The absorbance of each plasma sample was calculated as a fraction of the mean absorbance value of the positive controls after the background level of non-specific binding (absorbance of the negative controls) was subtracted from all samples. All samples were measured in duplicate, and deficiency of the CFHR1 protein was confirmed by western blot analysis using the rabbit polyclonal anti-CFHR1 antibody since CFHR1, CFHR2 and CFHR5 could be separated electrophoretically (Supplementary Material, Fig. S7).

The monoclonal JHD10 antibody is directed to the N-terminal region of CFHR1 but does not cross-react with factor H, CFHR3 or CFHR4. However, it does react with CFHR2 and to a small extent with CFHR5. This was accounted for in the ELISA by including duplicate homozygous CFHR3/CFHR1-deficient (CNP147) negative control samples on each plate, which measures the extent of cross-reactivity with other proteins (e.g. CFHR2, CFHR5). The absorbance of each plasma sample was, therefore, calculated after subtraction of this negative control value, as a fraction of the absorbance of the positive control value.

### Genotyping

A genome-wide association scan using 317 503 SNPs (HumanHap300 v1.0, Illumina) was carried out in 1004 individuals from a Croatian cross-sectional population survey in whom plasma CFH and CFHR1 had been measured, although only 308 144 SNPs and 941 samples passed quality control procedures as described (26). A second genome-wide association scan using 550 000 SNPs (Infinium HumanHap550-Duo BeadChip, Illumina) was carried out in 874 English cases of severe AMD (geographic atrophy, choroidal neovascularization) compared with 418 disease-free controls and scored using the BeadStudio v.3 software (Illumina). Additional genotypes in the EAMD case–controls and in an SAMD case–control series of 382 cases and 602 disease-free controls were scored using TaqMan probes (Applied Biosystems). Seven additional markers—rs1061170, rs1048663, rs412852, rs1066420, rs2019727, rs1410996 and the *CFHR3/CFHR1* deletion in the Croatian data set—and 11 markers in the Dutch (Rucphen) replication data set—rs1061170, rs6677604, rs1329428, rs7517126, rs4086175, rs3766404, rs1048663, rs412852, rs1066420, rs11582939, *CFHR3*/*CFHR1* deletion—were genotyped by TaqMan assay. *CFH* rs2274700 was genotyped by direct sequencing. Quantitative PCR (qPCR) of the *CFHR3/CFHR1* deletion polymorphism (18) was performed as described below. The non-synonymous *CFHR1* SNP rs388862 was genotyped by direct sequencing as described elsewhere (38).

### qPCR of *CFHR3/CFHR1* deletion polymorphism

Two sets of primers and minor groove binder probes (Applied Biosystems) were designed using the Primer Express v.2.0 software (Applied Biosystems). Each set consisted of a pair of primers and a 3′-fluorescent-tagged probe. One pair of primers and a FAM 5′-labelled probe were designed inside intron 3 of *CFHR3*:

- forward: TGGGCATTAGTCAAGAATACAGTAAAA
- reverse: ATTAATGCCGCTTCAATATGACTTT
- fluorescent probe: AATTAGAACACAATACTTGTTGGC.

Another pair of primers and a VIC 5′-labelled probe were designed inside the β-globin (*HBB*) gene and used as an endogenous control:

- forward: GGGCAGAGCCATCTATTGCTT
- reverse: TGGTGTCTGTTTGAGGTTGCTAGT
- fluorescent probe: TTGCTTCTGACACAACTG.

Each qPCR was carried out in triplicate using 384-well optical plates (Applied Biosystems). Five-microlitre reactions contained 10 ng of DNA, 2.5 μl of 2× TaqMan Universal PCR Master Mix (Applied Biosystems), 0.2 μl of each primer at 10 μm, 0.2 μl of each probe at 1 μm and 0.3 μl of water. Reactions were carried out using an ABI-HT7900 instrument (Applied Biosystems) as follows: 2 min at 50°C, 10 min at 95°C and 40 cycles of 15 s at 95°C and 1 min at 60°C. Fluorescence was read using the SDS software (Applied Biosystems) and the resulting *C*_t_ values were exported and analysed as described (52).

### Statistical analyses

Statistical tests were performed using SPSS (version 19), including Student's *t*-test, and *P*-values are indicated in the text and figures. Univariate and multivariate linear regression analyses were also performed using SPSS. Measurements were expressed as means ± standard errors of the mean (SEM) or as indicated.

#### Genome-wide association analysis

For genome-wide association analyses, additive effects of each locus were modelled in a linear regression of trait values on allele count, sex, age-by-sex and body mass index. For the Croatian sample, an additional polygenic random effect was fitted within a general linear mixed model to account for relatedness among individuals, and GWAS was carried out using the GenABEL R package (29,53). The effect size is reported per copy of the minor allele. From the Croatian data set, 27 SNPs including rs6677604 and neighbouring SNPs were carried over to a variance components analysis using SOLAR (54) to obtain estimates of additive genetic variance from the polygenic effect and the variance explained by each SNP.

Imputation using the multi-ethnic 1000 Genomes Phase I integrated variant set (release v3) ‘ALL’ reference panel (55,56) was done following guidelines from the GIANT consortium (http://genome.sph.umich.edu/wiki/IMPUTE2:_1000_Genomes_Imputation_Cookbook). Pre-phasing was performed using SHAPEIT2 (57) and imputation with IMPUTE2 (58,59).

### Instrumental variable (Mendelian randomization) analysis

An instrumental variable (Mendelian randomization) analysis was carried out (60–64) which exploits *CFH* and *CFHR1* genotypes as ‘instruments’ that randomize individuals to different concentrations of plasma CFH and CFHR1. This approach, which utilizes Bayesian models, has several advantages in the present context over classical (frequentist) methods (60). In particular, it is straightforward to combine information from different data sets and to allow for partially missing genotypes. The model makes three main assumptions: (i) the effects of genotype on AMD are not confounded (i.e. association is not due to a common underlying cause); (ii) the effects of genotypes on AMD are mediated only through their effects on plasma CFH or CFHR1 (no pleiotropy); (iii) there is no developmental compensation for the setting of the plasma CFH or CFHR1 level by genotype. If these assumptions hold, we can test for causal effects of CFH and CFHR1 by exploiting the genotypes as ‘instruments’ that perturb these biomarkers. Effect sizes on plasma CFH and CFHR1 are represented by linear regression coefficients, and effect sizes on AMD are represented by logistic regression coefficients. Further details are available in Supplementary Material.

## SUPPLEMENTARY MATERIAL

Supplementary Material is available at *HMG* online.

*Conflict of Interest statement*. None declared.

## FUNDING

This work was supported by grants from the Medical Research Council (UK) (A.F.W., N.D.H., J.R.W.Y., A.T.M., P.M.M.), Scottish Executive (Chief Scientist Office) (B.D., A.F.W.), Macula Vision Research Foundation (A.F.W.), Macula Disease Society (J.R.W.Y., A.T.M.), Wellcome Trust (H.C., I.R.), National Institute of Health Research (A.T.M.), Republic of Croatia Ministry of Science, Education and Sports grants to I.R. (108-1080315-0302), P.R.(196-1962766-2751), EU FP6 project EUROSPAN (Contract No. LSHG-CT-2006-018947), DeutscheForschungsgemeinschaft (DFG, Sk46) (C.S., P.F.Z.), Wellcome Trust Clinical Research Facility (Edinburgh, UK) for performing a genome-wide association scan. This research has received a proportion of its funding from the Department of Health's NIHR Biomedical Research Centre for Ophthalmology at Moorfields Eye Hospital and UCL Institute of Ophthalmology (J.R.W.Y.). The views expressed in the publication are those of the authors and not necessarily those of the Department of Health. The funders had no role in study design, data collection and analysis, decision to publish or preparation of the manuscript
